# Sociodemographic factors associated with dental students knowledge and attitudes regarding disinfection as a control measure to reduce the spread of COVID-19

**DOI:** 10.1038/s41598-025-86155-z

**Published:** 2025-01-17

**Authors:** Geanella Silva-Robles, Gissela Briceño-Vergel, Rosa Aroste-Andía, Emily Hernández-Huamaní, Marysela Ladera-Castañeda, Miriam Castro-Rojas, Percy Gavilán-Chávez, Luis Cervantes-Ganoza, César Cayo-Rojas

**Affiliations:** 1 School of Stomatology, Universidad Privada San Juan Bautista, Lima, Peru; 2 School of Stomatology, Universidad Privada San Juan Bautista, Ica, Peru; 3 Postgraduate School, Universidad Nacional Federico Villarreal, Research Group “Salud Pública-Salud Integral”, Lima, Peru; 4 Universidad Inca Garcilaso de la Vega, Doctorate in Public Health, Lima, Peru

**Keywords:** Associated factors, Attitudes, COVID-19, Dental education, Dental students, Disinfection, Knowledge, Diseases, Health care, Medical research

## Abstract

Despite maintaining a lower mortality rate and greater control of victims infected by COVID-19, the world’s population and science are still confronted with this coronavirus. Therefore, the aim was to assess the association between sociodemographic factors and the level of knowledge and attitudes of dental students regarding disinfection as a control measure to reduce the spread of COVID-19. This cross-sectional study evaluated 503 dental students from the capital city and one Peruvian province between February and June 2022. A validated 13-item questionnaire was used. A Poisson regression model with robust variance was used to evaluate the influence of the variables sex, age, year of study, marital status, place of origin, death of a family member due to COVID-19, and history of COVID-19, with the level of knowledge and attitudes of the dental students, considering a significance level of *p* < 0.05. Of the total participants, 14.3% showed sufficient knowledge, and 89.3% showed positive attitudes regarding disinfection as a control measure to reduce the spread of COVID-19. Furthermore, those from the capital city were 52% less likely to have sufficient knowledge regarding disinfection as a control measure to reduce the spread of COVID-19, compared to those from the province (APR = 0.48; 95% CI: 0.31–0.75). Moreover, none of the variables considered in this study were significantly associated with attitudes toward this topic (*p* > 0.05). A minority of dental students presented sufficient knowledge, while the majority presented positive attitudes regarding disinfection as a control measure to reduce the spread of COVID-19. In addition, being from the capital city was a limiting factor for sufficient knowledge. The variables sex, age, year of study, marital status, place of origin, death of a family member due to COVID-19, and history of COVID-19 were not influential factors for positive attitudes on this topic.

**Supplementary Information:**

The online version contains supplementary material available at 10.1038/s41598-025-86155-z.

## Introduction

The COVID-19 pandemic has led to changes in the occupational, social, economic, educational, and health fields^1^. The global case fatality rate for this infectious disease stood at 1.13% as of July 2022^2^. According to the report from 5 March 2023, there were a total of 676,609,955 confirmed cases and 6,881,955 deaths worldwide^3^. Furthermore, the World Health Organization has issued a warning that COVID-19 infections have increased by 52% in just one month, and this trend is expected to continue until 2024^4^. As a result, it is now a concern for health systems in all countries around the world^5^, despite advances in vaccination, diagnosis, and treatment methods^6^.

At the time of conducting this research, Peru was in the third wave of COVID-19 infection reaching historical peaks of infected people, for example, on 23 January 2022, 340,025 confirmed cases were reported causing 859 deaths, reaching a total of 1,479 deaths due to this disease on 6 February 2022^3^. Reports at the time indicated that omicron was the predominant variant across the country^7^. In parallel, the Ministry of Health (MINSA) reported that 67% of the general population had not yet received the third booster dose of the Covid-19 vaccine^8^.

Certain risks inherent to medical practice expose healthcare professionals. However, dentists have a higher risk of exposure to coronavirus due to their direct contact with saliva because, in clinical practice, they work in close contact with the oral cavities of patients, which further increases the risk by generating aerosols with rotating instruments or low- or high-speed turbines used in dental procedures^1,9–11^. It is because dental professionals and students are likely to be exposed to known or suspected sources of the SARS-CoV-2 virus during procedures that create bioaerosols^1,12^. This is why the Occupational Safety and Health Administration (OSHA) has put them in the “very high exposure risk” category. In addition, this pathogen can survive on solid surfaces exposed to body fluids from a few hours to several days^5,13,14^. It is evident that one of the effective strategies to halt or curtail the propagation of SARS-CoV-2 and other pathogens in the dental environment is to undertake a meticulous disinfection of hands, surfaces, medical equipment, and air^15^. This approach has been shown to play a pivotal role in mitigating the risk of cross-transmission of disease, thereby facilitating a more expeditious and secure recovery for patients, in addition to safeguarding the well-being of oral healthcare professionals. Furthermore, this approach has been shown to reduce the financial burden associated with medical complications, thereby enhancing the efficiency and quality of dental services^15,16^.

On the other hand, it is undeniable that COVID-19 affected the teaching-learning process and dental research, which forced dental schools to modify their traditional way of teaching to a remote teaching mode to adapt to the pandemic^17^. At the time of this study, universities in Peru were transitioning back to face-to-face teaching^18^. This posed a challenge for these institutions, particularly in adapting to clinical training, as it would be difficult to resume patient care while ensuring maximum protection against infection^1,17^. Therefore, stricter biosecurity standards, the use of personal protective equipment (PPE), and disinfection protocols were established to control and reduce the risk of COVID-19 infection^9^. This will help students identify the necessary measures and barriers to prevent further infections and the spread of the virus. It is important to understand the technical-scientific reasons behind these measures in order to increase awareness of the importance of prevention. This will not only save lives but also prevent cross-infection, especially for vulnerable people^9,19^. In view of the above, the aim was to assess the association between sociodemographic factors and the level of knowledge and attitudes of dental students regarding disinfection as a control measure to reduce the spread of COVID-19. The null hypothesis considered was that there are no sociodemographic factors associated with the knowledge and attitudes of dental students on this topic.

## Methods

### Study design

An analytical, cross-sectional, prospective, observational study was conducted. This report was written in accordance with the Strengthening the Reporting of Observational Studies in Epidemiology (STROBE) guidelines for observational studies^20^ and was conducted from February to June 2022 at the School of Stomatology of the Universidad Privada San Juan Bautista (UPSJB), based in Lima (Peruvian capital) and a branch in Ica (Peruvian province).

### Study population

**Fig. 1:**
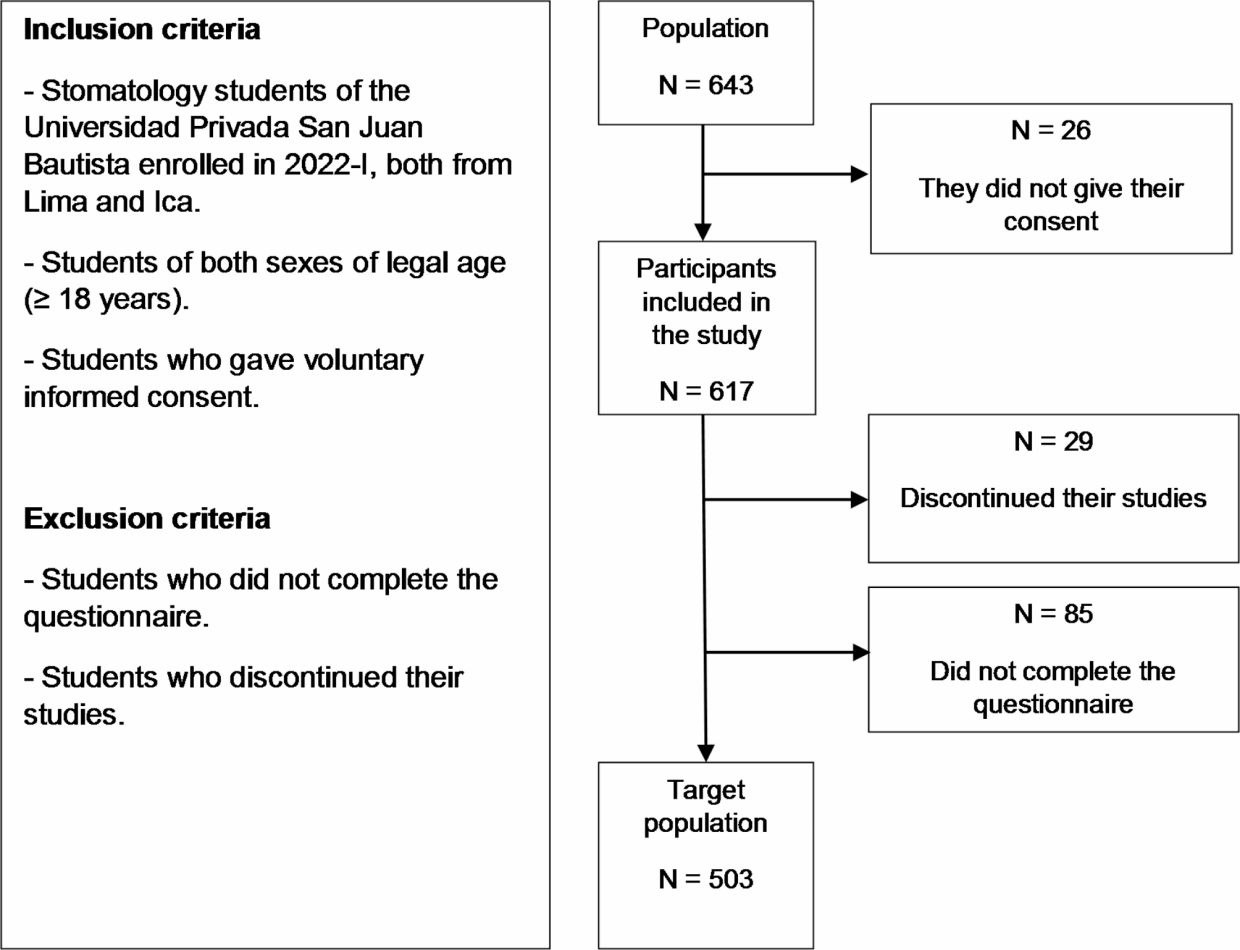
Participant selection flowchart.

The total population consisted of 643 students from the 1st to the 5th year of the professional career of Stomatology. The minimum sample size with EPIDAT 4.2 was 241 with the formula for estimating a proportion with finite population, considering a 95% confidence level, 5% estimation error and *p* = 50%. Therefore, in order to ensure the largest number of participants and to ensure statistical inference, it was decided to invite the entire population, provided that the total number of students was above the minimum required sample size, according to the eligibility criteria. Finally, the target population was *N* = 503 (93 1st year, 86 2nd year, 74 3rd year, 84 4th year, 123 5th year), with 299 participants from Lima (64 1st year, 42 2nd year, 37 3rd year, 56 4th year, 68 5th year) and 204 participants from Ica (29 1st year, 44 2nd year, 37 3rd year, 28 4th year, 55 5th year) **[**Fig. 1**].**

### Variables

The independent variables considered in this study were: gender^21^, and age^22^; and as possible confounding variables the year of study^23^, marital status^24^, place of origin^25^, death of a family member due to COVID-19^26^, and history of COVID-19^27^. While the dependent variables were knowledge and attitudes regarding disinfection as a control measure to reduce the spread of COVID-19.

### Instrument

A validated 13-question questionnaire^28^ with two dimensions, one on knowledge regarding disinfection as a control measure to reduce the spread of COVID-19 (K1-K10) (see supplementary material) and the other on attitudes regarding disinfection as a control measure to reduce the spread of COVID-19 (A1-A3), was used. Two English-speaking dentists translated and adapted the questionnaire into Spanish. Subsequently, three judges (a Doctor in Public Health and two researchers in Stomatology) with more than 15 years of experience validated the cross-culturality and the content of the instrument. They evaluated the relevance, objectivity, relevance, timeliness, sufficiency, clarity and methodology of the questionnaire, obtaining an acceptable Aiken’s V (V = 0.89; 95% CI: 0.85–0.92).

The knowledge dimension included 10 questions, K1 to K8 were single choice and the last two questions K9 and K10 were multiple choice. Each correct answer was assigned 1 point and an incorrect answer was assigned 0 points. The overall knowledge level was defined according to the following range: insufficient knowledge (< 7 points) / sufficient knowledge (≥ 7 points). The attitude dimension consisted of 3 questions (A1 to A3) with Likert scale responses being strongly disagree with 1 point, disagree with 2 points, neutral with 3 points, agree with 4 points and strongly agree with 5 points. The overall attitude was considered negative or unsatisfactory (< 10 points) / Positive or satisfactory attitude (≥ 10 points). To establish the optimal cut-off point for both dimensions, a three-level scale (low, medium and high) was made with the total scores based on Stanones’ rule [mean (x̄) ± 0.75 (standard deviation)], setting the cut-off point for knowledge at 7 points and for attitudes at 10 points. Furthermore, the accuracy of these cut-off points was validated using Livingston’s K^2^ coefficient, which resulted in 0.931 for knowledge and 0.915 for attitudes, which are acceptable values.

The internal consistency of the instrument was tested by Cronbach’s alpha reliability analysis, obtaining an (α) = 0.781 (95% CI: 0.711–0.840) for the knowledge dimension, and an (α) = 0.801 (95% CI: 0.722–0.860) for the attitude dimension. In addition, the concordance of scores was assessed in 100 randomly selected participants at two different times within 10 days, altering the order of the questions to avoid recall bias^29^, with the intraclass correlation coefficient (ICC) being very good for knowledge (ICC = 0.971; 95% CI: 0.957–0.981) and attitudes (ICC = 0.976; 95% CI: 0.964–0.984).

### Procedure

The questionnaire was distributed via institutional e-mails and personal social networks of each participant, via a link created in the virtual platform Google Classroom^®^. If no response was received, the invitation was repeated up to 3 times, with a frequency of once a week. The students’ informed consent to participate in the survey was at the beginning of the questionnaire, followed by the instructions for completing the questionnaire. However, they were free not to complete the questionnaire if they considered it appropriate. The online questionnaire was set up so that it could only be completed once with a maximum time limit of 10 min. In addition, they were asked to enter the initials of their first name, surname and age (example: GPSR23) to filter out repetitions in case someone accessed the link from two different email addresses. On the other hand, no students received incentives to participate in the study and had access to the questionnaire from February to June 2022. Only the principal investigator had access to the information collected and, to ensure confidentiality, it was stored on a digital device with a password. Finally, the results of the study were sent to whoever requested them via mail upon completion of the research.

### Statistical analysis

Data analysis was performed with the Statistical Package for the Social Sciences (SPSS) version 28.0. Descriptive statistics were applied to obtain absolute and relative frequency tables and bar charts. Pearson’s chi-square test was used for bivariate analysis and for expected values less than 5 Fisher’s exact test was used. For multivariate analysis, a Poisson regression model with robust variance was used. All analyses were performed, with significance set at *p* < 0.05.

### Ethical issues

The present study respected the bioethical principles of the Declaration of Helsinki related to confidentiality, respect, freedom and non-maleficence^30^. Approval was obtained from an institutional ethics committee of the Universidad Privada San Juan Bautista whose resolution was No. 8-2022-CIEI-UPSJB dated January 17, 2022. All methods were performed in accordance with the relevant guidelines and regulations. In addition, all participants gave their voluntary informed consent on the first page of the online questionnaire.

## Results

The response rate was 78.23% and the average age of the 503 participants was 24.5 ± 7.2 years. The majority were female with 62.8% of the total respondents. The most frequent age group was ≥ 22 years with 55.7%. The relative frequency of students by academic year ranged from 17.5 to 22.7%. Singles were the most predominant group with 88.3% of the total. In addition, 59.4% of the total number of participants were from the capital. Finally, 40.6% had lost at least one family member to COVID-19 and 51.5% had a history of having been ill with COVID-19 **[**Table 1**].**

**Table 1:** Sociodemographic characteristics of dental students.

Variable	Category	Frequency	Percentage
Gender	Male	187	37.2
	Female	316	62.8
Age group	< 22 years old	223	44.3
	≥ 22 years old	280	55.7
Year of study	1st year	93	18.5
	2nd year	88	17.5
	3rd year	102	20.3
	4th year	106	21.1
	5th year	114	22.7
Marital status	Single	444	88.3
	Married or cohabiting	59	11.7
Place of origin	Capital	299	59.4
	Province	204	40.6
Death of family member due to COVID-19	Yes	204	40.6
	No	299	59.4
History of COVID-19	Yes	244	48.5
	No	259	51.5

	Mean	SD	Median
Age	24.5	7.2	22.0

*SD* Standard Deviation.

**Fig. 2:**
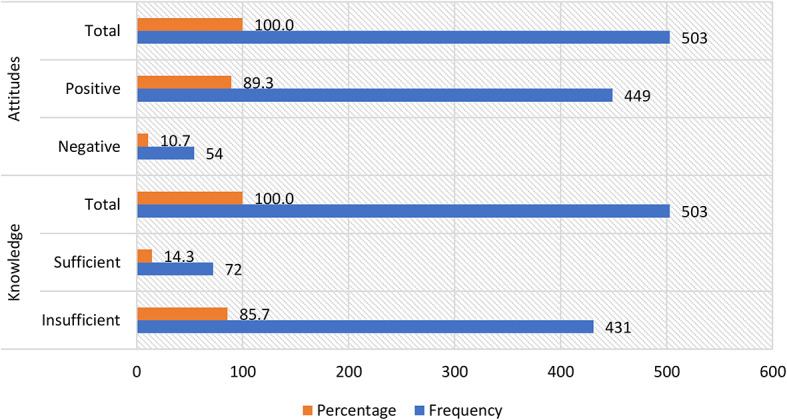
Absolute and relative frequency of knowledge and attitudes regarding disinfection as a control measure to reduce the spread of COVID-19 in dental students.

Of the 503 participants, 85.69% (95% CI: 82.38 − 88.99%) showed insufficient knowledge, while 14.31% (95% CI: 6.22 − 22.40%) showed sufficient knowledge; and finally, 10.74% (95% CI: 2.48 − 18.99%) showed negative attitudes, while 89.26% (95% CI: 86.40 − 92.13%) showed positive attitudes regarding disinfection as a control measure to reduce the spread of COVID-19 **[**Fig. 2**].**

Regarding knowledge of disinfection as a control measure to reduce the spread of COVID-19 in dental students, statistically significant age group associations were obtained with K1 (What is recommended to use to clean visibly soiled hands? ), K3 (How long can coronavirus remain infectious on inanimate surfaces? ) K7 (What is an effective hand disinfectant against coronavirus? ), K8 (What is the recommended disinfectant against coronavirus to disinfect waste before disposal? ) and K10 (What disinfectants and for how long should be applied to surfaces against coronavirus? ) (*p* = 0.028, *p* = 0.020, *p* < 0.001, *p* = 0.020 and *p* = 0.025; respectively). Gender was significantly associated with K5 (How long can SARS-COV-2 remain infective on stainless steel and plastic? ) and K8 (*p* = 0.049 and *p* = 0.009; respectively). Year of study was significantly associated with K1, K2 (What does the efficacy of a disinfectant against coronavirus depend on? ), K3 and K8 (*p* = 0.005, *p* = 0.047, *p* = 0.012 and *p* = 0.009; respectively). Marital status was significantly associated with K1, K6 (What should the disinfectants used against coronaviruses to disinfect floors, walls and dental arbitrary/operatory contain? ) and K7 (*p* = 0.015, *p* = 0.030 and *p* = 0.013; respectively); while place of origin was significantly associated with K7 (*p* < 0.001). In addition, having had at least one family member who died of COVID-19 was significantly associated with K1 and K10 (*p* = 0.019 and *p* = 0.028, respectively). Finally, having a history of COVID-19 was significantly associated with K1, K3 (*p* = 0.018 and *p* = 0.009; respectively) **[**Table 2**].**

**Table 2:** Knowledge regarding disinfection as a control measure to reduce the spread of COVID-19 in dental students.

Questions	Incorrect	Correct	Age group	Gender	Year of Study	Marital status	Place of origin	Family death by COVID-19	History of COVID-19
	f (%)	f (%)	*p	*p	*p	*p	*p	*p	*p
K1. What is recommended for cleaning visibly dirty hands?	168 (33.4)	335 (66.6)	0.028	0.140	0.005	0.015	0.720	0.019	0.018
K2. What does the efficacy of a disinfectant against coronavirus depend on?	64 (12.7)	439 (87.3)	0.661	0.954	0.047	0.833	0.990	0.794	0.798
K3. How long can the coronavirus remain infectious on inanimate surfaces?	435 (86.5)	68 (13.5)	0.020	0.463	0.012	0.423	0.911	0.150	0.009
K4. How long can SARS-COV-2 remain infectious on printing papers and tissue paper?	388 (77.1)	115 (22.9)	0.287	0.700	0.436	0.247	0.316	0.151	0.098
K5. How long can SARS-COV-2 remain infectious in stainless steel and plastic?	289 (57.5)	214 (42.5)	0.838	0.049	0.804	0.556	0.061	0.969	0.693
K6. What should the disinfectants used against coronaviruses to disinfect floors, walls and dental arbitrary/operatory contain?	202 (40.2)	301 (59.8)	0.122	0.194	0.620	0.030	0.467	0.066	0.857
K7. What is an effective hand sanitizer against coronavirus?	377 (75.0)	126 (25.0)	< 0.001	0.858	0.759	0.013	< 0.001	0.851	0.292
K8. What is the recommended disinfectant against coronavirus to disinfect waste before disposal?	173 (34.4)	330 (65.6)	0.020	0.008	0.009	0.211	0.080	0.545	0.266
K9. What are the recommended pre-procedural mouth rinses to reduce viral load?	335 (66.6)	168 (33.4)	0.632	0.763	0.241	0.064	0.349	0.349	0.299
K10. What disinfectants and for how long should be applied to surfaces against coronavirus?	281 (55.9)	222 (44.1)	0.025	0.225	0.064	0.052	0.101	0.028	0.094

**Based on Pearson’s chi-square (*p* < 0.05, significant association).

Regarding attitudes regarding disinfection as a control measure to reduce the spread of COVID-19 in dental students, statistically significant associations of age group were obtained with A1 (There is a high risk in a dental practice of becoming infected with SAR-CoV-2.) (*p* = 0.044). In addition, place of origin was significantly associated with A1 and A3 (To prevent COVID-19, it is important to disinfect frequently touched surfaces in a dental clinic) (*p* = 0.016 and *p* = 0.003; respectively). Finally, having had at least one family member die from COVID-19 was significantly associated with A2 (Following the disinfection guidelines will help in reducing the risk of becoming infected by SARS-CoV-2) (*p* = 0.001) **[**Table 3**].**

**Table 3:** Attitudes regarding disinfection as a control measure to reduce the spread of COVID-19 in dental students.

Questions	SD	D	*N*	A	SA	Age group	Gender	Year of Study	Marital status	Place of origin	Family death by COVID-19	History of COVID-19
	f (%)	f (%)	f (%)	f (%)	f (%)	*p	*p	*p	**p	*p	*p	*p
A1. There is a high risk in a dental practice of becoming infected with SAR-CoV-2.	22 (4.4)	24 (4.8)	57 (11.3)	154 (30.6)	246 (48.9)	0.044	0.882	0.244	0.332	0.016	0.504	0.785
A2. Following the disinfection guidelines will help in reducing the risk of becoming infected by SARS-CoV-2.	10 (2.0)	22 (4.4)	27 (5.4)	191 (38.0)	253 (50.3)	0.198	0.986	0.455	0.692	0.135	0.001	0.708
A3. To prevent COVID-19, it is important to disinfect frequently touched surfaces in a dental clinic.	9 (1.8)	27 (5.4)	22 (4.4)	143 (28.4)	302 (60.0)	0.205	0.744	0.803	0.113	0.003	0.636	0.620

*SD* strongly disagree, *D* disagree, *N* neutral, *A* agree, *SA* strongly agree.

*Based on Pearson’s chi-square (*p* < 0.05, significant association).

**Based on Fisher’s exact test (*p* < 0.05, significant association).

On the other hand, it could be observed that knowledge regarding disinfection as a control measure to reduce the spread of COVID-19, was significantly associated with the place of origin of the students (*p* = 0.002), while attitudes regarding disinfection as a control measure to reduce the spread of COVID-19 was significantly associated with having had at least one family member died from COVID-19 (*p* = 0.037) **[**Table 4**].**

**Table 4:** Sociodemographic factors in relation to knowledge and attitudes regarding disinfection as a control measure to reduce the spread of COVID-19 in dental students.

Variable	Category	Knowledge			Attitudes		
		Insufficient	Sufficient	p*	Negative	Positive	p*
		f (%)	f (%)		f (%)	f (%)	
Gender	Male	163 (32.4)	24 (4.8)	0.466	20 (4.0)	167 (33.2)	0.982
	Female	268 (53.3)	48 (9.5)		34 (6.8)	282 (56.1)	
Age group	< 22 years old	188 (37.4)	35 (7.0)	0.430	25 (5.0)	198 (39.4)	0.759
	≥ 22 years old	243 (48.3)	37 (7.4)		29 (5.8)	251 (49.9)	
Year of study	1st year	76 (15.1)	17 (3.4)	0.454	6 (1.2)	87 (17.3)	0.090
	2nd year	79 (15.7)	9 (1.8)		10 (2.0)	78 (15.5)	
	3rd year	85 (16.9)	17 (3.4)		18 (3.6)	84 (16.7)	
	4th year	94 (18.7)	12 (2.4)		8 (1.6)	98 (19.5)	
	5th year	97 (19.3)	17 (3.4)		12 (2.4)	102 (20.3)	
Marital status	Single	377 (75.0)	67 (13.3)	0.173	47 (9.3)	397 (78.9)	0.766
	Married or cohabiting	54 (10.7)	5 (1.0)		7 (1.4)	52 (10.3)	
Place of origin	Capital	268 (53.3)	31 (6.2)	0.002*	33 (6.6)	266 (52.9)	0.792
	Province	163 (32.4)	41 (8.2)		21 (4.2)	183 (36.4)	
Death of family member due to COVID-19	Yes	178 (35.4)	26 (5.2)	0.407	29 (5.8)	175 (34.8)	0.037*
	No	253 (50.3)	46 (9.1)		25 (5.0)	274 (54.5)	
History of COVID-19	Yes	211 (41.9)	33 (6.6)	0.624	27 (5.4)	217 (43.1)	0.817
	No	220 (43.7)	39 (7.8)		27 (5.4)	232 (46.1)	

*Based on Pearson’s chi-square (*p* < 0.05, significant association).

According to the crude Poisson regression model analysis with robust variance, knowledge (sufficient = 1 / insufficient = 0) and attitude (positive = 1 / negative = 0) regarding disinfection as a control measure to reduce the spread of COVID-19 were considered as dependent variables, and gender and age as independent variables, and year of study, marital status, place of origin, death of a family member due to COVID-19, and history of COVID-19 as possible confounding variables. When fitting the model with Poisson multiple regression with robust variance, it was observed that those in the capital city were 52% less likely to have sufficient knowledge regarding disinfection as a control measure to reduce the spread of COVID-19, compared to those in the province (APR = 0.48; 95% CI: 0.31–0.75). In addition, it was observed that none of the variables considered in this study, i.e., sex, age group, year of study, marital status, origin, death of family member by covid-19, and history of covid-19, were influential factors in positive attitudes regarding disinfection as a control measure to reduce the spread of COVID-19 in dental students (*p* > 0.05) **[**Table 5**].**

**Table 5:** Multivariable regression model of knowledge and attitudes regarding disinfection as a control measure to reduce the spread of COVID-19 in dental students.

Variable	Category	Knowledge								Attitudes							
		Crude model				*Adjusted model				Crude model				*Adjusted model			
		PR	95% CI		*p*	APR	95% CI		p*	PR	95% CI		*p*	APR	95% CI		p*
			**LL**	**UL**			**LL**	**UL**			**LL**	**UL**			**LL**	**UL**	
Gender	Male	0.85	0.56	1.33	0.468	0.79	0.50	1.26	0.319	1.00	0.94	1.07	0.982	0.99	0.93	1.06	0.807
	Female	*Ref.*				*Ref.*				*Ref.*				*Ref.*			
Age group	< 22 years old	1.19	0.78	1.82	0.430	0.92	0.55	1.52	0.743	0.99	0.93	1.05	0.760	0.99	0.91	1.07	0.733
	≥ 22 years old	*Ref.*				*Ref.*				*Ref.*				*Ref.*			
Year of study	1st year	1.23	0.66	2.27	0.516	1.36	0.68	2.73	0.384	1.05	0.96	1.14	0.290	1.06	0.96	1.17	0.266
	2nd year	0.69	0.32	1.46	0.330	0.66	0.30	1.45	0.298	0.99	0.90	1.09	0.851	1.00	0.90	1.11	0.971
	3rd year	1.12	0.60	2.07	0.724	1.13	0.60	2.11	0.710	0.92	0.83	1.03	0.138	0.93	0.83	1.05	0.221
	4th year	0.76	0.38	1.51	0.434	0.85	0.42	1.69	0.635	1.03	0.95	1.12	0.440	1.04	0.96	1.14	0.356
	5th year	*Ref.*				*Ref.*				*Ref.*				*Ref.*			
Marital status	Single	1.78	0.75	4.24	0.192	1.71	0.70	4.17	0.239	1.02	0.92	1.12	0.775	1.01	0.91	1.12	0.796
	Married or cohabiting	*Ref.*				*Ref.*				*Ref.*				*Ref.*			
Place of origin	Capital	0.52	0.34	0.79	0.003	0.48	0.31	0.75	0.001*	0.99	0.93	1.05	0.790	0.98	0.92	1.05	0.518
	Province	*Ref.*				*Ref.*				*Ref.*				*Ref.*			
Death of family member due to COVID-19	Yes	0.83	0.53	1.30	0.409	0.85	0.55	1.32	0.468	0.94	0.88	1.00	0.048	0.94	0.88	1.01	0.053
	No	*Ref.*				*Ref.*				*Ref.*				*Ref.*			
History of COVID-19	Yes	0.90	0.59	1.38	0.624	0.90	0.60	1.37	0.631	0.99	0.93	1.06	0.817	1.00	0.95	1.07	0.842
	No	*Ref.*				*Ref.*				*Ref.*				*Ref.*			

*APR* prevalence ratio adjusted under Poisson regression model with robust variance, *95% CI* 95% Confidence Interval, *LL* Lower Limit, *LS* Upper Limit.

*Adjusted multiple regression model (*p < 0.05, significant association).

## Discussion

Infection control remains one of the most important challenges in health care delivery worldwide, including dental schools/colleges, as the adoption of infectious disease prevention and control measures in clinical settings are crucial to provide a safe environment for students, patients, educators, and health care professionals^5,31^. Surface disinfection is one of the aspects to which most attention should be paid, together with biosafety measures and the use of appropriate protective elements^32^. In this sense, it is important for students to have the necessary knowledge about disinfection protocols in order to guarantee adequate performance in the clinical area. Therefore, the aim of this research was to evaluate the association of sociodemographic factors with the level of knowledge and attitudes regarding disinfection as a control measure to reduce the spread of COVID-19 in dental students, so due to the results obtained, the null hypothesis had to be rejected.

The results of this study indicated that 85.7% of the students presented insufficient knowledge regarding disinfection as a control measure to reduce the spread of COVID-19. These results differ from those reported by Pathak et al.^33^, who found that overall more than 60% of dental students in India considered temperature control and hand disinfection as necessary measures to prevent the spread of COVID-19. In addition, they found that liquid soap with water or an alcohol-based hand sanitiser > 60% could be the most effective form of hand hygiene, they also found that sodium hypochlorite was the most effective way of disinfecting surgical surfaces, and finally they considered the use of a mouthwash by the patient prior to treatment to be necessary. This discrepancy could be due to the fact that the present research was conducted in the year 2022, which probably led students to lower their guard or downplay the importance of disinfection protocols against COVID-19^34^, as they probably felt safe with the third dose of the coronavirus vaccine^35^, which may have led them to neglect acquiring and/or updating their knowledge on this topic. In contrast, the study by Pathak et al.^33^ was conducted in the year 2020, a time when students had not been vaccinated and therefore used to update their knowledge and adopt precautionary measures according to accredited daily news, webinars, online education programme and published scientific articles, among others^33^.

On the other hand, place of origin was found to be significantly associated with knowledge of the most effective hand sanitiser against coronavirus. This could perhaps be attributed to the fact that students from the provinces, having information about the exacerbated increase in cases of COVID-19 in the Peruvian capital^36^, sought further training on which disinfectants work as an effective alternative for hand cleaning to prevent the spread of the disease^37^. In this regard, WHO recommended rubbing hands with an alcohol concentration of 60–80% after hand washing for adequate disinfection^38^.

With regard to attitudes, it was observed that 89.3% of the students had positive attitudes regarding disinfection as a control measure to reduce the spread of COVID-19. This indicates that students have good intentions to put it into practice, since as future health professionals they have an obligation and responsibility to decrease the risk of contagion derived from health care work, encouraging them to have positive attitudes^39^, even without having sufficient knowledge to implement disinfection guidelines against COVID-19^28^. This agrees with Umeizudike et al., who reported that half of the Nigerian dental students had sufficient knowledge of COVID-19, although 95.1% had positive attitudes towards infection control^40^.

While it is true that in this study, the bivariable analysis showed that attitudes regarding disinfection as a control measure to reduce the spread of COVID-19 were significantly associated with having at least one sick family member. However, in a multivariable regression model, it was not considered to be an influential factor, showing that the association of variables does not always necessarily indicate causality or influence^41,42^. Therefore, this finding may indicate that having a sick family member was not a determining factor in having a good attitude towards this issue, as dental students may have felt that the responsibility and commitment to prevent infection was inherent in their profession with good clinical practice, regardless of the risk of exposing a vulnerable family member^43,44^.

On the other hand, a novel finding of the present study, under a multivariate statistical analysis model, was that students in the capital were 52% less likely to have sufficient knowledge regarding disinfection as a control measure to reduce the spread of COVID-19 compared to those in the provinces. These results could be due to the fact that, at the time of the study, students from the provinces felt the need to be more informed and updated, due to the fear they felt when they heard on the news that there was a considerable increase in COVID-19 cases in the Peruvian capital^36^, and even more so knowing that if this had happened to them, the situation would have become very critical, as health centres in the Peruvian provinces still did not have sufficient medical staff and infrastructure to deal with the disease^45^. Furthermore, it was observed that none of the variables considered in this study, i.e., gender, age group, year of study, marital status, origin, death of a family member due to covid-19, and history of covid-19, were associated or influential factors in the positive attitudes regarding disinfection as a control measure to reduce the spread of COVID-19 in dental students. This could be attributed to the fact that more than 80% of all participants, according to the variables mentioned, presented positive attitudes, perhaps because the students were in similar conditions, as they were all in the process of adapting to face-to-face classes and already had protocols established by the university and the Ministry of Health for disinfection, biosecurity, and preventive measures for patient care^11,46^. It is also likely that the gaps between the categories of each variable were closed, with respect to positive attitudes, because the vast majority of students had received the third dose of the COVID-19 vaccine^1^.

Unlike other health science students, dental students are at an increased risk of infection due to the nature of their clinical training. This is because they work in the oral cavity of patients and are frequently exposed to contaminated bioaerosols, saliva, blood, and other body fluids^31,47^. In order to provide patients with safe and effective dental treatment, infection control is of utmost importance. Research has shown that coronaviruses can persist on surfaces for varying lengths of time, ranging from a few hours to several days^5,13,14^, depending on factors such as the nature of the surface, humidity, and temperature^14^. To control the spread of the virus, it is essential for students to recognize the threat and maintain strict disinfection protocols. They should also use personal protective equipment and take necessary measures during their dental practice^5,48,49^.

Among the limitations of the present research, we can mention that it was not possible to survey students in person, because the education sector was in the process of adapting to face-to-face teaching, nor was it possible to monitor knowledge about pre-pandemic, pandemic, and post-pandemic disinfection in order to assess the variation and durability of this knowledge over time. Another limitation of this research is that a thoroughly validated instrument was not used; therefore, it is essential to validate its metric properties in future research to ensure its validity and reliability in a Latin American context. This will allow its applicability not only to COVID-19 but also to any cross-infection disease. Finally, it is recognized that, although these results cannot be extrapolated to the whole country, they can nevertheless be a starting point for developing studies in this line of research in order to identify gaps in knowledge and take appropriate measures to plan and reinforce training in disinfection as a means of controlling and reducing COVID-19 in professional dental practice.

It is recommended that university authorities, academic coordinators, and teachers re-evaluate and update disinfection protocols for infection control, as well as carry out training on this topic so that students can adequately develop their clinical practices and be prepared to continue to face COVID-19, since as of March 2023 it continues to claim lives, with 216,000 deaths reported daily worldwide and 87 deaths daily in Peru^3^. Furthermore, at present (January 2024), according to the Peruvian Ministry of Health, a cumulative case fatality rate of 4.85% and 220,632 deaths have been reported since the beginning of the pandemic^50^. On the other hand, research on cross-infection control and mitigation in dental students following a pandemic is recommended, as well as educational interventions on this topic to improve knowledge and attitudes in dental practice in the face of changes in public health policies, new scientific evidence, or possible epidemics or pandemics.

## Conclusion

A minority of dental students had sufficient knowledge, while the majority had positive attitudes regarding disinfection as a control measure to reduce the spread of COVID-19. In addition, being from the capital city was a limiting factor for sufficient knowledge. The variables sex, age, year of study, marital status, place of origin, death of a family member from COVID-19, and history of COVID-19 were not influential factors for positive attitudes on this topic. It is recommended that university authorities, academic coordinators, and teachers re-evaluate and update disinfection protocols so that students are prepared to face possible epidemics or pandemics. In addition, it is necessary to design and validate a standardized instrument to effectively assess knowledge and attitudes toward COVID-19 and other cross-infections adapted to the Latin American context.

## Electronic supplementary material

Below is the link to the electronic supplementary material.

Supplementary Material 1

## Data Availability

All data analyzed during this study are available from the corresponding author on reasonable request (cesarcayorojas@gmail.com).

## Abbreviations

APR: Adjusted Prevalence Ratio
CI: Confidence interval
COVID-19: COronavirus DIsease 2019
OSHA: Occupational Safety and Health Administration
PPE: Personal Protective Equipment
PR: Prevalence Ratio
STROBE: STrengthening the Reporting of OBservational studies in Epidemiology
SPSS: Statistical Package for the Social Sciences
