# Supplementary material for: Sociodemographic factors associated with dental students knowledge and attitudes regarding disinfection as a control measure to reduce the spread of COVID-19
Source: Sci Rep. 2025 Jan 17;15:2280. doi: 10.1038/s41598-025-86155-z (PMC11742444; doi:10.1038/s41598-025-86155-z)
Supplement: Supplementary file 1 — Supplementary Material 1 [file 41598_2025_86155_MOESM1_ESM.docx]

Supplementary Material: Knowledge Questionnaire

| Item | Responses |
| --- | --- |
| **K1.** What is recommended for cleaning visibly dirty hands? | Soap and water for 20 s |
|  | 70–85% Alcohol-based hand rub (ABHR) |
|  | Soap and water for 20 seconds and then alcohol-based hand rub |
|  | Don’t Know |
| **K2.** What does the efficacy of a disinfectant against coronavirus depend on? | Composition of a disinfectant |
|  | Use of more than one disinfectant at a time |
|  | Contact time of disinfectant on the surface |
|  | Don’t Know |
| **K3.** How long can the coronavirus remain infectious on inanimate surfaces? | 3 days |
|  | 6 days |
|  | 9 days |
|  | Don’t Know |
| **K4.** How long can SARS-COV-2 remain infectious on printing papers and tissue paper? | 30 min |
|  | 2 h |
|  | 3 h |
|  | Don’t Know |
| **K5.** How long can SARS-COV-2 remain infectious in stainless steel and plastic? | 1 day |
|  | 7 days |
|  | 14 days |
|  | Don’t Know |
| **K6.** What should the disinfectants used against coronaviruses to disinfect floors, walls and dental arbitrary/operatory contain? | 1000 mg/L Chlorine |
|  | 2 mg/L Ozone |
|  | 6 mg/L Peracetic Acid |
|  | Don’t Know |
| **K7.** What is an effective hand sanitizer against coronavirus? | 60% to 70% Alcohol-based hand sanitizer |
|  | 75% to 80% Alcohol-based hand sanitizer |
|  | Alcohol-free hand sanitizer |
|  | Don’t Know |
| **K8.** What is the recommended disinfectant against coronavirus to disinfect waste before disposal? | Hydrogen peroxide |
|  | Chloroxylenol (Dettol) |
|  | Sodium hypochlorite (bleach) |
|  | Don’t Know |
| **K9.** What are the recommended pre-procedural mouth rinses to reduce viral load? | 1.5% Hydrogen peroxide |
|  | 0.23% to 7% Povidine-Iodine (PVP I) |
|  | 0.5% Chlorhexidine |
|  | Don’t Know |
| **K10.** What disinfectants and for how long should be applied to surfaces against coronavirus? | 0.1% Sodium hypo-chloride, 0.5% hydrogen peroxide within 1 min |
|  | 0.1% Sodium hypo-chloride and 42–61% ethanol within 20 s |
|  | 0.02% Chlorhexidine di-gluconate within 2 min |
|  | Don’t Know |
